# The Use of the Thermometer in the Diagnosis of Fever

**Published:** 1870-08

**Authors:** Thos. C. Murphy

**Affiliations:** Green Valley, Ill.


### ARTICLE III.—*The Use of the Thermometer in the Diagnosis of Fever.* By THOS. C. MURPHY, M.D., Green Valley, Ill.

Galen defined fever as a preternatural heat. Other additional clauses have since been added, such as quick pulse, turbid urine, etc. But with the advance of science, many of the high-sounding terms have been swept away. Yet, to-day preternatural heat cannot be blotted out. As during fever there always is an increase of temperature, such increase of heat must arise from an increased tissue-change, and have its immediate cause in alterations of the nervous system.

Now it is plain to see, that the hand is not the proper instrument with which to measure the amount of abnormal heat. A man may shake with a paroxysm of ague until his teeth chatter, he will get as near to the fire as is convenient, in fact he is *cold*. But the thermometer will indicate an increase of heat during the cold stage of from six to eight degrees Fahrenheit. Now, when the physician uses his hand to judge of the amount of abnormal heat, or when the feelings of the patient alone are taken as the measure of his temperature, it can easily be understood how such kind of observation must be doubtful, fallacious and unsatisfactory. The use of the thermometer as a clinical assistant was recognized over one hundred years ago. But at that time it seemed impossible to find a reliable instrument. Now the thermometer is within the reach of all, and the time taken to apply it is so insignificant that the physician or student of clinical medicine has no excuse for neglecting to use it. The careful physician counts the pulse and respirations in all cases of sickness, and it is now incumbent on him to measure the heat. I claim that the use of the thermometer facilitates the clinical recognition of disease, and the progressive physician should use all means at his command to find out "what's the matter," and lack of time is no excuse, for patients soon learn to attend to the instrument, if they are not too sick. What physician is not able to spare his patients from three to five minutes extra time? The less such persons a community is blessed with, the better for them.

The form of thermometer I prefer is the curved one, and the axilla the best situation to place the instrument in, taking care that all the clothing is removed from the axilla. The axis of vision should fall perpendicular on the column of mercury in the tube. In all observations of the temperature note the pulse and number of respirations at the same time.

The temperature of the body is the result of the opposing action of two factors. 1st. Of the development of heat from the chemical changes of the food, and by the conversion of mechanical force into heat, or by direct absorption from without. Opposed to this is evaporation, which assists in equalizing the heat of the body.

Our best information in regard to the normal heat of the body comes from Valentin, Bernard, Edwards, and Dr. John Davy, of England. All agree that the heat varies in different parts of the body. But they also agree that the normal heat of the body, in completely sheltered parts of the surface, is 98° or 98$^{5}$° Fahrenheit. A temperature of 99° or a depression below 97$^{5}$° Fahrenheit are sure indications of disease, if the increase or depression is persistent.

As a general rule, an increase of temperature of one degree above 98° Fahrenheit, corresponds with an increase of ten beats of the pulse per minute, if the patient is not under the influence of sedatives. Increase of temperature is always present during fever; we would not have fever if such were not the case. The greater the amount of heat (as measured by the thermometer) the greater destructive tissue-change there is going on. Coldness of the surface is a delusive symptom. I claim for the physician who depends on the use of the thermometer as a guide, greater certainty in his diagnosis and prognosis. The physician should use the thermometer daily, and in all cases, and, if possible, make the observation at the same hour, in the most important cases at least. If called to a patient who has been in good health previous to the attack, should you find the temperature at 105° or 106° Fahrenheit, you may feel safe in saying, the case is of malarious origin. On the other hand, should a patient's temperature range for several days above 98$^{5}$° as follows: 1st day, morning, 99° Fahrenheit; evening, 100°; 2d day, morning, 99$^{5}$°, evening, 106$^{5}$°—morning, 101$^{5}$°, evening 102$^{5}$°; 3d day, morning, 102°, evening, 103°; 4th day, morning, 103°, evening, 103$^{5}$° to 104° Fahrenheit, you may diagnose typhoid fever, even should the usual symptoms of typhoid be absent. But should the rise of temperature be abrupt, to 104° and fall to 98° within the 24 hours, you have not to do with a case of typhoid fever. Constant, persistent elevation of temperature, is as certain of the history of typhoid fever, as that of the sun's setting in the west. Certain febrile diseases are found to give typical ranges of temperature, or daily fluctuations throughout their course. This is the case in uncomplicated cases. Malarious fever, typhus, typhoid fever, small-pox, scarlatina, measles, rheumatism, pneumonia. In each of the above diseases the temperature is one of the most certain means for determining the real state, as regards morbid disturbances and complications, and a careful observation of the temperature from day to day, is indispensable for judging as to the prognosis. Frequently it affords the best and ultimate means of deciding in doubtful cases, and often it is the best corrective of a too hasty conclusion: for should complications be liable to occur, the increase of temperature will put you on your guard.

A case, recently in my own practice, will serve to illustrate. A case of pneumonia, on the morning of the 4th day gave a temperature of $102^{5}$° Fahrenheit; case doing well, but on the morning of the 5th day I find the temperature 105°; on calling at night, I find a true diphtheritic exudate on the fauces. I am positive there was no exudate in the morning, and there were no symptoms of diphtheria on the morning of the 4th day. If it were not for the aid of the thermometer in the above case, the patient might have been gathered to his fathers.

Again, in typhoid fever, if you find during the third week of a severe case that the temperature has fallen to 99° or $99^{5}$°, the reparative stage has begun—the healing of Peyer's patches and a similar fall of temperature at night, shows convalescence has commenced. The morbid process does not end until the normal temperature of the body returns and remains unchanged through the day and night.

I do not wish any one to think that the thermometer will do all for them, it will not; it is only a useful and trusty aid to the physician, and student. Don't ride it. What would we say of the man who objects to auscultation or percussion? In auscultation we have the advantage that our ears are always here. In percussion the hand is always at hand. We cannot say the same of the hand as a heat measurer, for it don't tell the truth, it fails us here at least, even if " feeling is the naked truth." The live man of to-day must bring to his aid the improvements of the times, seek to elevate himself in his profession, feel that he is interested, and leave the American Medical Association to talk of reform, make stump speeches, everything only what they claim to do—elevate the profession.

It is no reason that because, perchance, our grandfathers did not use the thermometer we should not—it won't do. Times have changed since Eberle wrote. Calomel and Jalapa are not recognized, to-day, as panaceas for all ills. Progress marks the march of scientific medicine. We must not puff about reform, but assist in doing it.

